# Purification and characterization of a novel antibacterial peptide against Clostridium perfringens

**DOI:** 10.1099/mic.0.001573

**Published:** 2025-07-07

**Authors:** Alex Novodvorski, Avalene Kong, Hai Yu, Dion Lepp, Ashley Brott, Jason Carere, Stephen Seah, Joshua Gong

**Affiliations:** 1 Guelph Research and Development Centre, Agriculture and Agri-Food Canada, Guelph, Ontario, N1G 5C9, Canada; 2 Department of Molecular and Cellular Biology, University of Guelph, Guelph, Ontario, N1G 2W1, Canada; 3 Food Research Division, Bureau of Chemical Safety, Health Canada, Ottawa, Ontario, K1A 0K9, Canada

**Keywords:** antibacterial peptide, antimicrobial spectrum, *Bacillus velezensis*, *Clostridium perfringens*, SH3 domain

## Abstract

*Bacillus velezensis* HG88 was isolated from ileal mucosa samples of egg layer hens that were raised without the use of antibiotics. Its cell-free supernatant (CFS) was found to inhibit the growth of *Clostridium perfringens*, the causative agent of necrotic enteritis in chickens. The inhibitory compound was determined to be proteinaceous due to its susceptibility to protease digestion. The antimicrobial activity was specific towards *C. perfringens*, as the CFS did not inhibit the growth of Gram-positive or Gram-negative bacteria across nine different species and two yeast fungi. Separation of proteins from the CFS followed by peptide mass fingerprinting and genomic analyses of the strain enabled the identification of a putative antibacterial peptide with an export signal for secretion from the cell. The peptide from *B. velezensis* HG88, named IP_HG88_, has sequence similarity to bacterial SH3 domains that are known to bind to the peptide portion of peptidoglycan. The gene encoding this peptide was cloned, and the peptide was purified from recombinant *Escherichia coli* as an N-terminal His-tagged peptide. The IP_HG88_ with or without the His-tag inhibited the growth of *C. perfringens* with a minimum bactericidal concentration of ~57.0 or 39.1 µg ml^−1^, respectively. The 3D structure of IP_HG88_ was also predicted using AlphaFold 2.0.

## Data Availability

All data generated from the current study are included in this article. The datasets used and/or analysed during the study are available from the corresponding author upon request.

## Key points

1. Selected *B. velezensis* HG88 specifically antagonizes *C. perfringens.*
2. The purified antibacterial peptide (IP_HG88_) has retained the activity.
3. IP_HG88_ appears to be novel with a single SH3 domain.

## Introduction

Necrotic enteritis (NE) is an economically important gut disease in poultry, resulting in an estimated US$6 billion in annual losses to the global poultry industry [1]. The disease is caused by *Clostridium perfringens* type G strains [2], which has been historically controlled by antibiotics in feed. *C. perfringens* type G is characterized by producing NetB toxin that is a key factor in NE pathogenesis [3]. With the global migration towards antibiotic-free production of poultry, it is urgent to develop effective non-antibiotic technologies to control NE disease. Probiotic bacteria, including *Bacillus*, have long been used to control enteric infections in poultry [4]. For example, a *Bacillus subtilis* strain, PB6, that was isolated from a healthy chicken gut and shown to mitigate *C. perfringens* induced NE in broilers when used as a dietary supplement, reducing intestinal lesion scores and * C. perfringens* counts in the gut [5,6]. Compared with *Lactobacillus* bacteria (a group with established use as probiotics) that are sensitive to heat and require protection, *Bacillus* bacteria have intrinsic resistance to adverse environmental factors, since they are able to form spores and produce multiple digestive enzymes, both increasing protection and animal digestibility [7,8]. *Bacillus* bacteria also produce bacteriocins, a group of thermostable antibacterial peptides. Bacteriocins exert antimicrobial activities to specifically inhibit various types of bacteria, including pathogens, providing a competitive advantage to bacteriocin-producing bacteria in diverse environments [9]. The aforementioned PB6 strain produced an antibacterial peptide of 960–983 Da in size that inhibited the growth of *C. perfringens* ATCC 13124 and was resistant to degradation under high temperatures (100 and 121 °C) and to trypsin digestion, due to the presence of lipid or carbohydrate moieties that modified the peptide [5]. Resistance to proteolytic enzymes found in poultry gut is important to determine, as the secretion of protease enzymes such as trypsin in the chicken gut for the degradation of dietary proteins poses a pressure on antimicrobial peptides [10,11]. The historically safe use of bacteriocins has been well recognized as a strategy against infectious diseases [12]. Bacteriocins from *Bacillus* that can antagonize *C. perfringens* have also been reported, e.g. sublancin from *B. subtilis* 168 [13,14]. However, sublancin has broad spectrum activity against other Gram-positive bacteria [15]. To discover more bacteriocins with novel properties, the present study has isolated and characterized a *Bacillus* strain (*Bacillus velezensis* HG88) and an antibacterial peptide (IP_HG88_) it produces, which specifically inhibits the growth of *C. perfringens* CP1, a type G strain, aiming to provide an additional tool to control NE in poultry. This peptide contains a single SH3 domain; this domain has not been previously associated with antimicrobial properties.

## Methods

### Micro-organisms and culture preparation

*C. perfringens* CP1 was originally isolated from an NE case in Ontario [16]. *Escherichia coli* DH5*α* and *Escherichia coli* LOBSTR-BL21 (DE3) were purchased from Thermo Fisher Scientiﬁc (Burlington, ON, Canada) and Kerafast Inc. (Boston, MA, USA), respectively, which were used for gene cloning and expression. In the experiment to determine the antimicrobial spectrum, additional bacteria, *Escherichia coli* K-12 ATCC 25253, *Salmonella typhimurium* PT193 strain SA982424 [17], *Bifidobacterium animalis* CGI1, *Bifidobacterium pullorum* CGI2, *Lactobacillus johnsonii* CFM8, *Lactobacillus gallinarum* CFM20, *Lactobacillus crispatus* CGM19, *Enterococcus faecalis* CGI3 and *Enterococcus cecorum* CGM68, and fungi, *Pichia pastoris* X33 (Thermo Fisher Scientiﬁc) and *Sporobolomyces* ATCC 20291 that is classified as *Sporobolomyces salmonicolor*, were used as target micro-organisms. All the strains of *Bifidobacterium*, *Lactobacillus* and *Enterococcus* stated above were isolated from chicken gut or faecal samples by the research group at Guelph Research and Development Centre, Agriculture and Agri-Food Canada. The strain identities were determined by 16S rDNA sequence similarity analysis with blast. All media or media components were purchased from Thermo Fisher Scientific. Media for bacterial growth include Brain Heart Infusion (BHI) and Columbia Agar supplemented with 5% defibrinated sheep blood for *C. perfringens*, lysogeny broth (LB) or agar for *Bacillus*, *Escherichia coli* and *Salmonella*, De Man–Rogosa–Sharpe (MRS) for *Lactobacillus* and *Enterococcus* and MRS++ (modified MRS by adding 0.2 g Na_2_CO_3_, 0.1 g CaCL_2_.2H_2_0 and 10 ml of 5% filter sterilized l-cysteine HCL monohydrate to 1 l of MRS) for *Bifidobacterium*. Fungi *P. pastoris* and *Sporobolomyces salmonicolor* were grown on yeast extract peptone dextrose broth or agar aerobically (shaking at 180 r.p.m.) at 30 °C for 16 h. All the bacteria were cultured at 37 °C for 16–24 h depending on their growth rates. While *Bacillus*, *Escherichia coli* and *Salmonella* were cultured aerobically with shaking at 180 r.p.m., *C. perfringens*, *Bifidobacterium*, *Lactobacillus* and *Enterococcus* were incubated under an anaerobic atmosphere (5% hydrogen, 10% CO_2_ and 85% N_2_).

*B. velezensis* HG88 was isolated from an ileal mucosa sample of Rhode Island Red egg layer hens in Ontario, Canada, that were raised without the use of antibiotics. It was selected after heat treatment (80 °C, 10 min) of the mucosal sample and examination of its antimicrobial and protease activity. The protease activity was determined by spotting the HG88 culture (10 µl) on a proteinase activity selecting agar medium and measuring the size of digestion zones after 24-h incubation at 37 °C. The selected medium contains 25 g soy protein, 1.07 g NaH_2_PO_4_, 1 g Na_2_HPO_4_, 0.5 g MgSO_4_, 0.01 g FeSO_4_ and 15 g agar per litre. The protein and the salts with agar were prepared and autoclaved separately before the two parts were mixed in an equal volume when they were cooled down to 80 °C. To prepare the cell-free supernatant (CFS) of HG88 culture, a 48-h-grown culture in LB (37 °C with shaking at 180 r.p.m.) was centrifuged (10,000 ***g***, 10 min) to pellet the bacterial cells followed by filtration of the supernatant through a 0.22 µm polyethersulfone membrane.

### Antimicrobial assays

A micro-dilution assay was used to determine antimicrobial activities. Briefly, to assess the antimicrobial activity towards * C. perfringens*, the HG88 CFS, semi-purified or purified recombinant protein was added to an assay mixture that was subjected to a series of twofold dilutions followed by incubation at 37 °C for up to 24 h under an anaerobic atmosphere. The assay mixtures had a 1% final concentration of the *C. perfringens* inoculum and BHI broth at 1× concentration. The assay was conducted in Costar clear bottom 96-well plates with 200 µl of the assay mixture per well. Each assay had a negative control (no CP1 inoculum included), and a positive control that contained a CP1 inoculum but no CFS, semi-purified native protein or purified recombinant protein. The growth of *C. perfringens* was monitored by measuring the OD at *λ*=600 nm via a plate reader (Biotek; Agilent Technologies, Mississauga, ON, Canada). All the samples showing inhibition of the pathogen growth in the 96-well plates were plated onto the Columbia Agar with sheep blood and incubated at 37 °C for up to 24 h under an anaerobic atmosphere to confirm survival or death of the cultures, from which the minimum bactericidal concentration (MBC) of tested agents towards *C. perfringens* was determined. All the assays were conducted in triplicate.

The micro-dilution assay was also used to determine the antimicrobial spectrum of the HG88 CFS in the same manner as described above with modifications to the temperature, time of incubation and aerobic or anaerobic conditions, for optimal growth of the target micro-organisms. The growth (OD_600nm_) of cultures in the broth of the media described above was measured, and the cultures were plated onto the agar plates of the media after the assay to verify the viability of the cultures.

### Protease digestion

To determine the protein nature of the antimicrobial activity, four different proteases including trypsin, chymotrypsin, pepsin and proteinase K were used to digest CFS or purified proteins, of which trypsin, chymotrypsin and pepsin are endogenous enzymes produced by the chicken digestive system. Lyophilized trypsin, chymotrypsin, proteinase K and pepsin (Millipore-Sigma, Oakville, ON, Canada) were dissolved, individually, in 20 mM HEPES at pH 7.5. Trypsin and chymotrypsin were added to samples (CFS or purified proteins) at a final concentration of 10 µg ml^−1^, while protease K and pepsin were added at a final concentration of 0.3 µg ml^−1^. All the samples were digested for 24 h at 37 °C followed by incubation in a 100 °C heat block for 1 min to stop the digestion.

### Protein separation

The HG88 CFS was concentrated from 4 l to 40 ml using a 400 ml Amicon stirred cell containing a 76 mm regenerated cellulose Ultrafiltration Disc filter with a 3 kDa nominal molecular weight limit. Both the concentrated CFS and flow-through were tested for antimicrobial activity. Concentrated CFS was buffer exchanged with 20 mM HEPES at pH 7.5 before cation exchange chromatography using Source 15S resin on an ÅKTA Explorer 100 apparatus (GE Bioscience, Baie d’Urfé, QC, Canada). The mobile phase was 20 mM HEPES at pH 7.5 with a gradient increase from 0 to 1 M NaCl over 10 CV. Fractions of 5 ml were collected over the elution time.

### DNA sequencing and analysis

To prepare DNA samples, a 16-h-grown HG88 culture (1.5 ml, 37 °C) was extracted using the GenElute Bacterial Genomic DNA Kit (Millipore-Sigma). Samples were prepared for Nanopore sequencing using the SQK-RBK004 Rapid Barcoding Kit (VWR, Mississauga, ON, Canada) according to the manufacturer’s instructions. Briefly, DNA was diluted to 400 ng in water and mixed with 2.5 µl of barcode fragmentation mix. Samples were loaded on a thermocycler at 30 °C for 1 min and 80 °C for 1 min to bind DNA before cooling on ice. Barcoded DNA was purified with AMPure beads and washed with 70% EtOH. The MinION Mk1B flow cell was primed using kit reagents. A rapid adapter was added to the DNA samples before the addition of the sequencing buffer and loading beads. The treated DNA library was added to the primed FLO-MIN106 flow cell and sequenced using a MinION Mk1B for 48 h at −180 mV. After sequencing, basecalling and demultiplexing were performed with guppy v 6.5.7. The resulting Fastq files were quality-trimmed using PoreChop v 0.2.4 and assembled with Unicycler v 0.4.8 to produce a single contig. Gene prediction and annotation was performed with Prokka v 1.14.6 (https://github.com/tseemann/prokka). Whole-genome sequencing (WGS) was also performed on a NextSeq 550 instrument (Illumina, San Diego, CA, USA) using 300-cycle NextSeq 500/550 High Output Kit v2.5 (Illumina). Paired-end 2×150 bp sequencing libraries were prepared using the NEBNext® Ultra™ II FS DNA Library Prep Kit for Illumina (New England BioLabs, Whitby, ON, Canada) according to the manufacturer’s instructions. Species identification was performed on raw reads with Kraken 2 [18] using the Standard Kraken 2 database (downloaded 9 September 2024). The web-based tools DMSZ Type strain genome server (https://tygs.dsmz.de/) and KmerFinder 3.2 (https://cge.food.dtu.dk/services/KmerFinder/) were also used to classify species based on whole-genome sequences.

### Peptide fingerprinting and analysis

Proteins were analysed by SDS-PAGE consisting of a Tris-tricine dual buffer system in a Bio-Rad Mini-protean vertical electrophoresis cell. The 16% acrylamide resolving gel was run at 20 mA and 200 V for 2 h according to the method reported by Schägger [19]. PageRuler low-range unstained protein ladder was used as the molecular weight (M_W_) marker (Thermo Fisher Scientific). Following Coomassie blue staining, a band was excised from the SDS-PAGE and destained with 50 mM ammonium bicarbonate and 50% acetonitrile (ACN). The clear gel was then dried with 100% ACN, and peptides were reduced with 10 mM DTT and alkylated in 55 mM iodoacetamide before overnight digestion with a mix of trypsin and endoproteinase Lys-C. To stop digestion, 5% formic acid in 50% ACN was added to the extracted peptides, followed by vacuum centrifugation to dry the sample before liquid chromatography-tandem MS analysis.

The Vanquish Neo UHPLC system was coupled with Orbitrap Exploris 240 mass spectrometer (Thermo Fisher Scientific) using the Easy-Spray source for nano Liquid chromatography-mass spectromtery (LC-MS) protein identification. Peptides were first trapped and washed on a Pepmap Neo C18 trap column (5 µm, 300 mm × 5 mm) and then separated on EASY-Spray columns (75 µm I.D. ×50 cm) with a maximum pressure of 1,200 bar. The nano LC-MS system was controlled with Standard Instrument Integration (SII) for Xcalibur software. The mobile phase A and weak wash liquid was water with 0.1% formic acid, and the mobile phase B and strong wash liquid was 80% ACN with 0.1% formic acid. The gradient was as follows: 4–19% B over 72 min, 19–29% B over 28 min, 29–45% B over 20 min and a 14.5-min wash at 100% B with a flow rate of 300 nl min^−1^. The autosampler temperature was 7 °C, and the column temperature was 45 °C. The sample was injected with fast loading set to ‘enabled’ with pressure control at 500 bar. The column Fast Equilibration function was set to ‘enabled’ with pressure control at 800 bar, and the equilibration factor was set to 3. Vial bottom detection was set to ‘enabled’.

The Orbitrap Exploris MS was operated in data-dependent acquisition (DDA) mode using a full scan with m/z range of 375–1,500, Orbitrap resolution of 60,000, normalized target value of 300% and maximum injection time set to auto. The intensity threshold for the precursor was set to 1×104. MS/MS spectra starting from 120 m/z were acquired in DDA mode with a cycle time of 2 s, where the precursors were isolated in a window of 1.6 Da and subsequently fragmented with higher-energy collisional dissociation using a normalized collision energy of 30%. Orbitrap resolution was set to 15,000. The normalized AGC target was set to standard, and the maximum injection time was set to auto.

Raw data were analysed using PEAKS Online X build 1.8.2023-03-01_092215 (Bioinformatics Solutions Inc., Waterloo, ON, Canada). PEAKS DB was set up for *de novo* sequencing to search the *B. velezensis* HG88 protein sequence database produced from Nanopore sequencing created with the Nanoseq pipeline with a parent mass tolerance of 10 p.p.m. and fragment mass tolerance of 0.02 Da. The following variable modifications were considered: oxidation of methionine and deamidation of asparagine and glutamine. Cysteines were all considered to be carbamidomethylated. The results were filtered to a less than 1% false discovery rate.

### Cloning of HG88_03711 antibacterial peptide

The gene HG88_03711 encoding the antibacterial peptide (referred from hereon as IP_HG88_, i.e. inhibitory peptide HG88) was identified through blast alignment of the protein sequences from peptide fingerprinting analysis against HG88 genome sequence. The gene was amplified by PCR using genomic DNA extracted from strain HG88 and three sets of primers (Table 1). The PCR products were digested with restriction enzymes corresponding to the restriction sites introduced into the primers and ligated into similarly digested plasmid vectors, pET28a, pET30a or pCG004 [20]. The ligation mixtures were transformed into *Escherichia coli* DH5*α*, and the plasmids from recombinant *Escherichia coli* were extracted and sequenced to confirm the presence of the correct inserts. *Escherichia coli* LOBSTR-BL21 (DE3) (Kerafast Inc., Shirley, MA, USA) and *B. subtilis* 1A976 [21] were used for gene expression, respectively. The constructs made with pET28a and pET30a were aimed at expressing IP_HG88_ in the cytoplasm (i.e. without signal peptide) and periplasm (i.e. with signal peptide) of *Escherichia coli* LOBSTR-BL21 (DE3), respectively. The pET28a and pET30a plasmids append a hexahistidine tag (His-tag) at the N- and C-terminus of the recombinant protein, respectively, and were selected on the nutrient agar containing kanamycin (50 µg ml^−1^). Full-length HG88_03711 ligated to the plasmid pCG004 was used to test the expression in *B. subtilis* 1A976.

**Table 1.** PCR primers for amplifying gene HG88_03711 to insert into the indicated respective plasmids. Introduced NdeI, HindIII and BsaI sites are bolded

Plasmid	Sequence (5′-3′)
pET28a	F: GGG G**CA TAT G**GC AGC TTT CAA ACC TAA AGC TG
	R: GGG G**AA GCT T**TT ATA AAC CGT AAT AAT AAG ATA GTG TGG TG
pET30a	F: CGG G**CA TAT G**GT ACG TCG TTT GTC GAT C
	R: GGG G**AA GCT T**TA AAC CGT AAT AAT AAG ATA GTG TGG TG
pCG004	F: CCC C**GG ATA GAG ACC** TTG GTA CGT CGT TTG TCG ATC
	R: CCC C**GA ACT GAG ACC** TTA TAA ACC GTA ATA ATA AGA TAG TGT GGT G

### Purification of the recombinant antibacterial peptide

*Escherichia coli* LOBSTR, transformed with the pET28a vector encoding the antibacterial peptide (IP_HG88_), was grown (37 °C, 180 r.p.m.) in 4×1 l LB containing 50 µg ml^−1^ kanamycin. When the culture reached an OD_600nm_ of 0.6±0.05, 1 mM of isopropyl ß-D-1-thiogalactopyranoside (IPTG) was added, and the culture was grown at 15 °C with shaking for 16 h. The culture was centrifuged at 15,680 ***g*** for 20 min, and pelleted cells were re-suspended in 20 mM HEPES at pH 7.5 buffer and lysed by French Press at 15,000 p.s.i. The crude cell lysate was centrifuged twice at 39,191 ***g*** for 10 min to remove the insoluble cell debris. The cell-free extract was passed through a 0.20 µm nylon syringe ﬁlter and incubated for 1 h at 4 °C with Ni^2+^-NTA resin in 50 mM phosphate buffer (pH 8.0) containing 20 mM imidazole and 300 mM sodium chloride. The mixture was poured into a gravity column and washed with the same buffer. The His-tagged protein was then eluted using the same buffer containing 150 mM imidazole. The eluted protein was exchanged into 20 mM sodium HEPES at pH 7.5 buffer by dilution into a stirred cell with a YM3 ﬁlter (Amicon, Miami, FL, USA).

Thrombin was used to cleave the His-tag at a final concentration of 1 NIH U ml^−1^ in 50 mM sodium phosphate and 10% glycerol. To remove thrombin, p-aminobenzamidine agarose was added before the mixture was incubated with Ni^2+^-NTA resin to remove uncleaved His-tagged protein. Pure cleaved protein was collected from the mixture supernatant and buffer exchanged into 20 mM HEPES at pH 7.5 using a stirred cell with a YM3 ﬁlter (Amicon). Fractions were analysed on SDS-PAGE, and protein concentrations were determined using a bicinchoninic acid kit (Thermo Fisher Scientific) following the manufacturer’s instructions.

### Protein structure prediction of IP_HG88_

To obtain the predicted tertiary structures of IP_HG88_, the aa sequence was input into AlphaFold 2.0, an AI protein structure prediction tool developed by DeepMind, as described previously [22,23]. The generated PDB file was viewed using PyMOL, in which high predicted local distance difference test (pLDDT) value areas were coloured in blue and the trypsin cutting sites were visualized in magenta on yellow.

## Results

### Antimicrobial activity and spectrum of HG88 CFS

The HG88 CFS completely inhibited the growth of *C. perfringens* CP1 on Columbia Agar with sheep blood after 24-h co-incubation with the pathogen at 37 °C. In addition, the strain demonstrated protease activity, generating a clear digestion zone (5 mm in diameter) on the agar plate containing soy proteins.

The antimicrobial activity of HG88 CFS towards 12 different bacterial and fungal species, including *C. perfringens*, *Escherichia coli*, *Salmonella typhimurium*, *P. pastoris*, *Sporobolomyces salmonicolor*, two species of *Bifidobacterium* or *Enterococcus* and three species of *Lactobacillus*, was tested. *C. perfringens* exhibited no growth after co-incubation with the CFS that was diluted from 2-fold to 16-fold (Table 2). However, no inhibition in growth by the CFS towards other bacterial and fungal species was observed.

**Table 2.** Antimicrobial spectrum of CFS from *B. velezensis* HG88. The assays were conducted in triplicate. −, No microbicidal activity; +, microbicidal activity (no growth). Fourfold and 8-fold dilutions of the CFS were also tested, and they showed the same results as those at 2-fold and 16-fold dilutions

Target microbe	Microbicidal activity		
	2× dilution	16× dilution	32× dilution
*C. perfringens* CP1	+	+	−
*Escherichia coli* K-12 ATCC 25253	−	−	−
*Salmonella typhimurium* PT193	−	−	−
*Bifidobacterium animalis* CGI1	−	−	−
*Bifidobacterium pullorum* CGI2	−	−	−
*L. johnsonii* CFM8	−	−	−
*L. gallinarum* CFM20	−	−	−
*L. crispatus* CGM19	−	−	−
*Enterococcus faecalis* CGI3	−	−	−
*Enterococcus cecorum* CGM68	−	−	−
*P. pastoris* X33	−	−	−
*Sporobolomyces* ATCC 20291	−	−	−

While the heat treatment and digestion with trypsin or chymotrypsin had no effect on the antimicrobial activity of HG88 CFS towards *C. perfringens* (~100% activity retained), protease K digestion abolished the activity, implying that a peptide could be responsible for the antimicrobial activity.

### Purification and identification of the antibacterial peptide

Whole-genome sequencing of HG88 was performed using both short-read and long-read technologies to characterize the strain and facilitate the identification of the antibacterial peptide-encoding gene. blast analysis of the 16S rRNA gene sequence revealed that the strain shared more than 99% sequence identity to *Bacillus amyloliquefaciens* in the NCBI nr database. Species identification based on the alignment of WGS reads with Kraken 2 revealed that 74.7% of total reads aligned to the *Bacillus* genus, with the majority of species-level reads aligning to *B. velezensis* (10.07%), followed by *B. amyloliquefaciens* (0.84%) and *Bacillus siamensis* (0.11%). The DMSZ Type Strain Genome Server classified HG88 as *B. velezensis*, whereas KmerFinder 3.2 classified it as *B. amyloliquefaciens*. A phylogenetic tree based on WGS alignment showed that HG88 clustered more closely with *B. velezensis* than *B. amyloliquefaciens*, although the two species are closely related (data not shown).

**Fig. 1.**
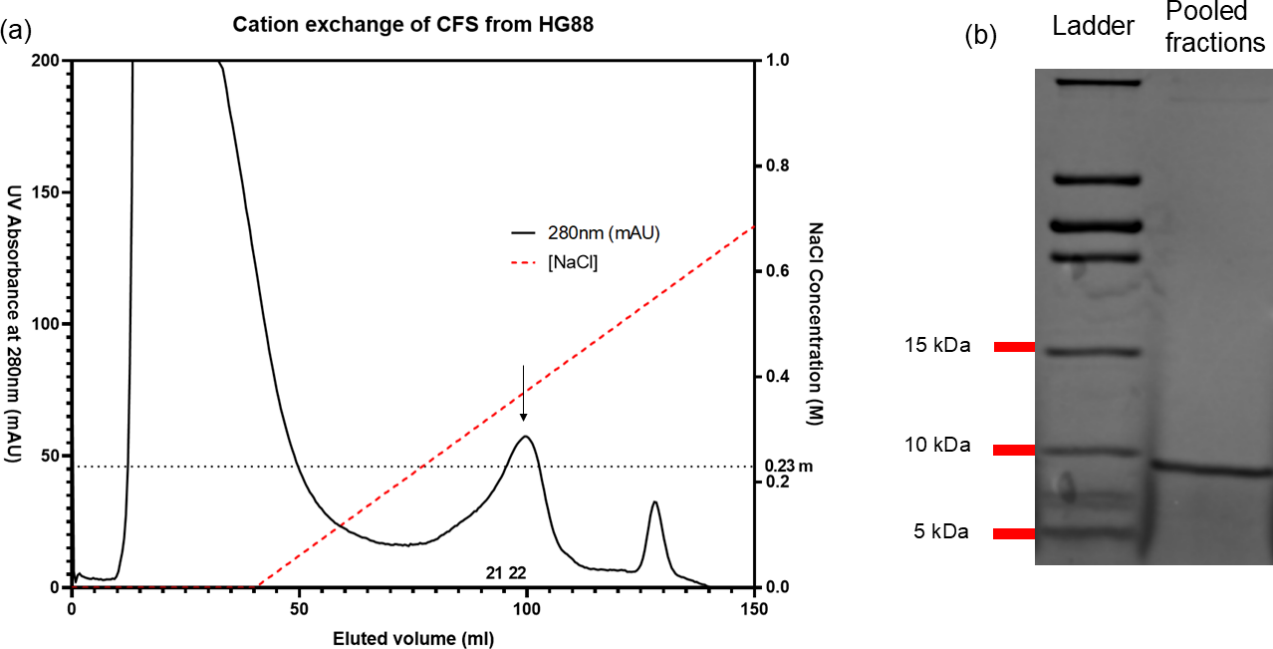
Partial purification of an antibacterial peptide from the CFS of *B. velezensis* HG88. (**a**) FPLC chromatogram showing cation exchange chromatography of the CFS. Fractions 21 and 22 eluted at 0.23 M NaCl, showed antimicrobial activity towards *C. perfringens* CP1 and had an MBC of ~632.5 µg ml^−1^. (**b**) Tris-tricine SDS-PAGE of pooled fractions 21 and 22, showing a band consisting of the protein of interest with the M_W_ of ~9 kDa.

Fractionation of the concentrated HG88 CFS was achieved by cation exchange chromatography (Fig. 1a). Fractions that were eluted with buffer containing ~0.23 M NaCl exhibited antibacterial activity towards *C. perfringens* and were thus pooled. The pooled fractions demonstrated a major protein band in the SDS-PAGE gel with a M_W_ of ~9 kDa (Fig. 1b). The semi-purified antibacterial peptide was examined for its antimicrobial activity, and the MBC was ~690 µg ml^−1^.

**Fig. 2.**
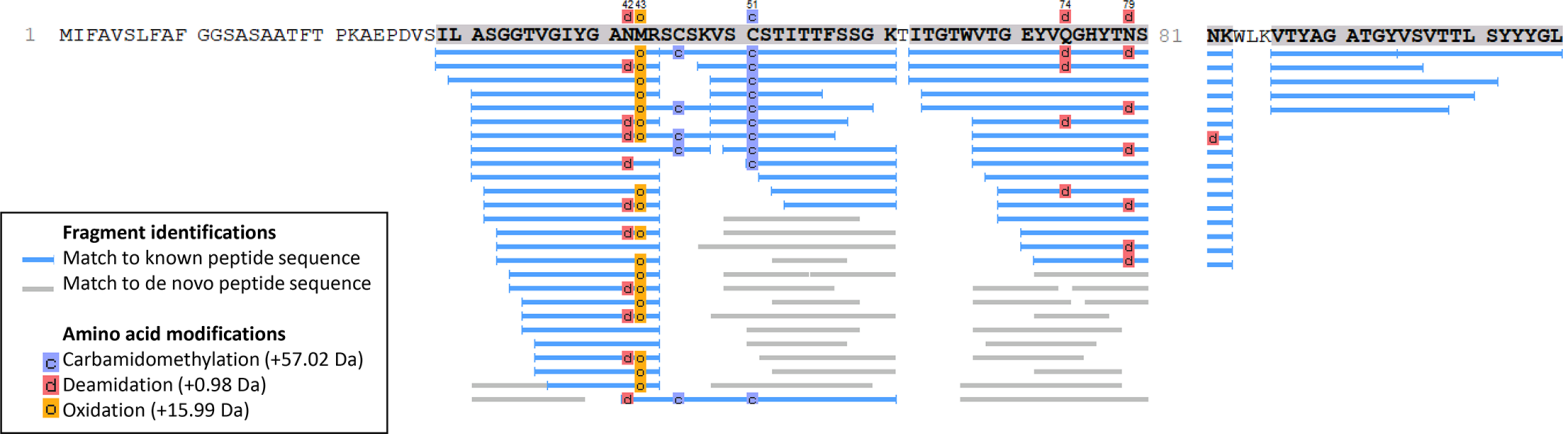
Protein fragment coverage map generated through LC-MS peptide fingerprinting. Results from MS analysis of the in-gel digested antibacterial peptide that is shown in Fig. 1(b). A protein with a theoretical size of 12.34 kDa was identified through matching 39 peptides with 64% coverage of the HG88_03711 gene.

**Fig. 3.**
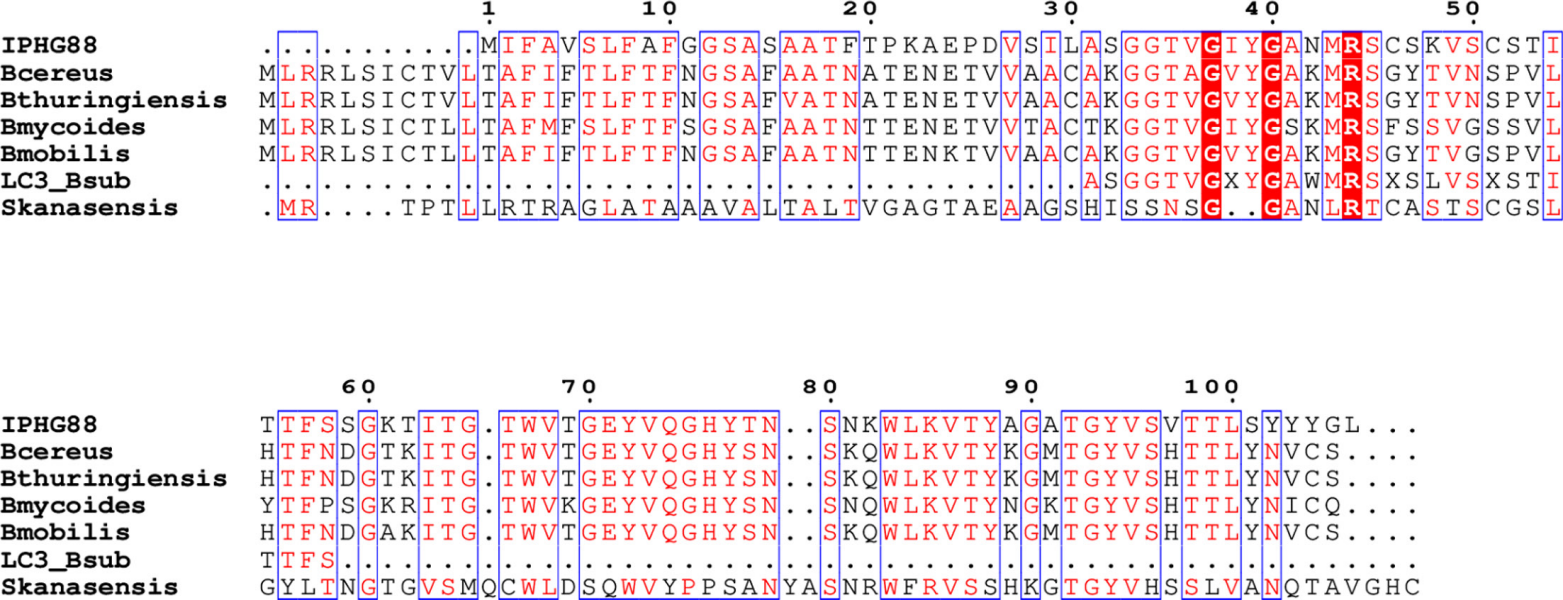
Alignment of IP_HG88_ sequence with homologues. IP_HG88_ was aligned to the partial sequence of LC3 from *B. subtilis* (LC3_Bsub, UniProt Q9R5C6) and full sequences from *Bacillus mycoides* (GENBANK WP_044439908.1), *Bacillus cereus* (GENBANK MCU5489889.1), *Bacillus thuringiensis* (GENBANK WP_098361274.1), *Bacillus mobilis* (GENBANK WP_098237633.1), *Bacillus mycoides* (GENBANK WP_044439908.1) and *Streptomyces kanasensis* (GENBANK KUH40362.1) using clustalx and then displayed in ESPript 3.0 [33]. Residue numbering for IP_HG88_ is shown on top of the alignment. Partially conserved residues are boxed, and the residues are shown in red fonts while fully conserved residues are highlighted in red.

To identify the antibacterial peptide, the single major protein band in the SDS-PAGE gel was excised and then subjected to tryptic peptide MS fingerprinting. Thirty-nine unique peptides with a 64% coverage of a hypothetical protein HG88_03711 from * B. velezensis* HG88 were found (Fig. 2). The encoded protein, IP_HG88_, contains a signal peptide corresponding to the first 27 aa as predicted by SignaIP-6.0 [24]. This signal peptide sequence was not detected from the peptide mass fingerprinting consistent with its cleavage upon secretion to the CFS; hence, the 64% coverage did not include the signal peptide. blast search of the NCBI database using the full-length protein sequence revealed the presence of homologues in a number of *Bacillus* genomes that have been annotated as hypothetical proteins, SH3 domain-containing proteins or anti-fungal peptides. IP_HG88_ also shows sequence similarity to a partial sequence of a protein known as LC3 (UniProt accession number Q9R5C6). A sequence alignment of IP_HG88_ with LC3 and other homologues is shown in Fig. 3.

### Expression, purification and characterization of recombinant IP_HG88_

**Fig. 4.**
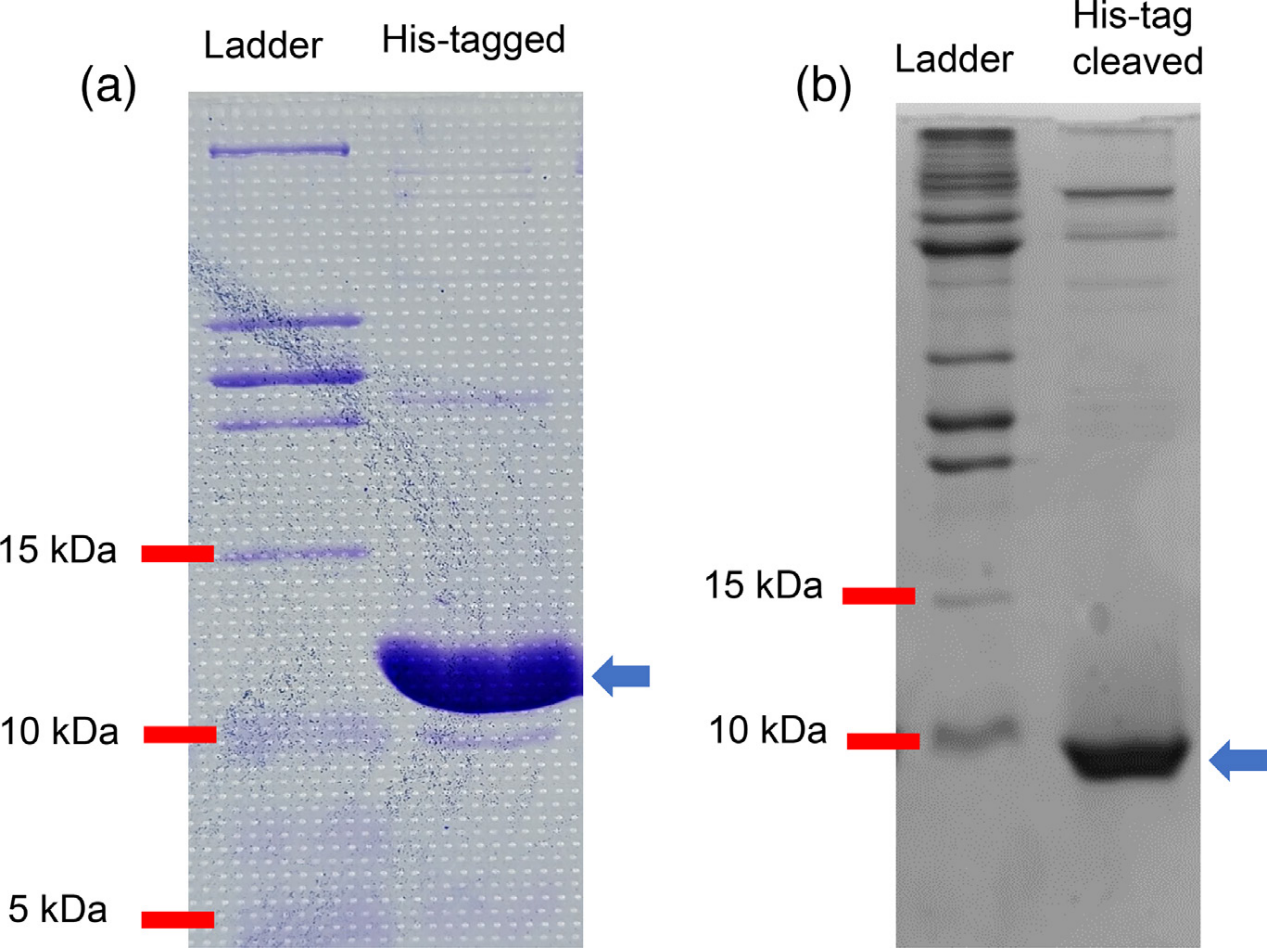
Tris-tricine SDS-PAGE of IP_HG88_. (**a**) His-tagged IP_HG88_ produced recombinantly in *Escherichia coli* LOBSTR and purified using a Ni^2+^-NTA column. Arrow pointing to the most prominent band at >10 kDa (the predicted M_W_ of the peptide is 11.8 kDa). (**b**) Purified IP_HG88_ with cleavage of the His-tag by thrombin. Arrow pointing to the most prominent band at <10 kDa close to the predicted M_W_ of 9.47 kDa.

The recombinant peptide (IP_HG88_) was not detected in the periplasm of *Escherichia coli* LOBSTR-BL21 (DE3) or in the culture supernatant of *B. subtilis* 1A976. However, an N-terminal His-tagged peptide produced from the construct lacking the signal peptide in the expression vector pET28a was successfully purified from the cytoplasm of the recombinant *Escherichia coli* (Fig. 4a). The yield of the peptide from 2 l of the culture was 10.7 mg. The His-tagged peptide showed a band of ~12.5 kDa, and the His-tag (encoded in the pET28a plasmid) was cleaved by thrombin, yielding a peptide with a M_W_ slightly less than 10 kDa as determined by SDS-PAGE (Fig. 4b).

**Fig. 5.**
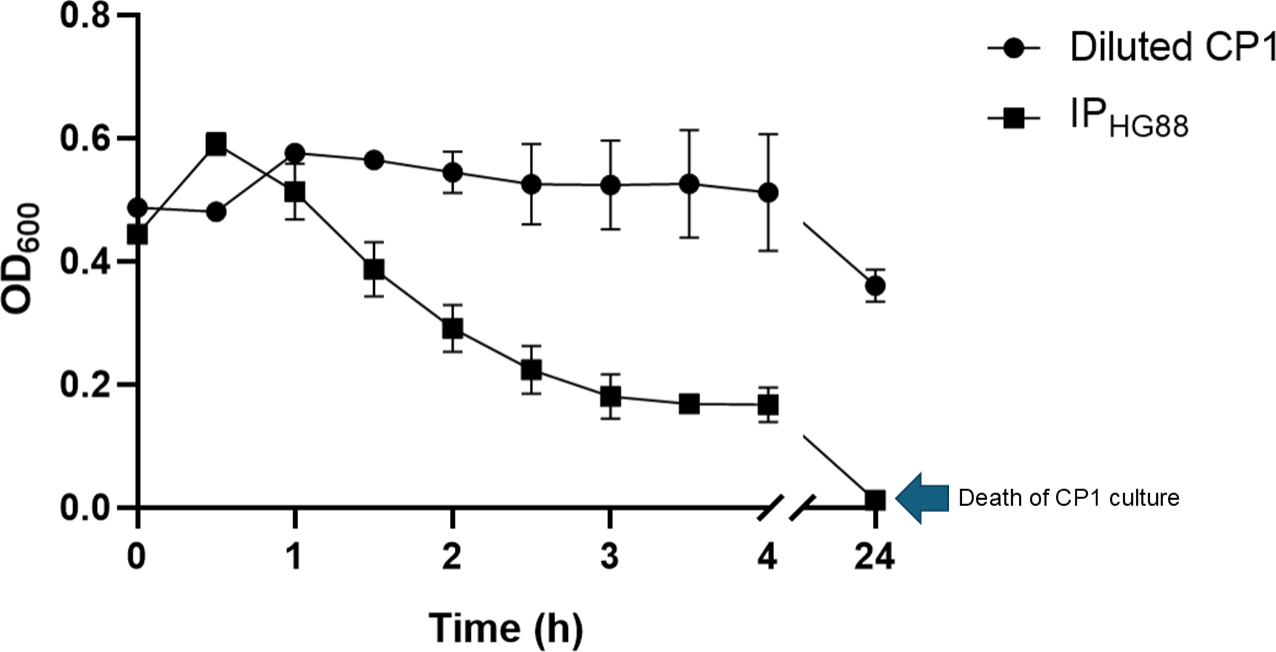
Growth of *C. perfringens* CP1 in the presence of His-tagged IP_HG88_. *C. perfringens* CP1 was grown anaerobically until the late logarithmic phase with 20 mM HEPES buffer (diluted CP1) or IP_HG88_ (final concentration: 211 µg ml^−1^) added at 0 h of the assay.

The antibacterial activity towards *C. perfringens* of the N-terminal His-tagged IP_HG88_ was examined. A decrease in the pathogen growth (OD_600nm_) was observed starting at 1 h and decreasing to zero by the end of the assay (24-h incubation) in the presence of IP_HG88_ (Fig. 5). The *C. perfringens* culture could not be recovered by plating on Columbia Agar with sheep blood after the 24-h incubation. The MBC of IP_HG88_ with or without the His-tag was determined to be ~57.0 and 39.1 µg ml^−1^, respectively.

**Fig. 6.**
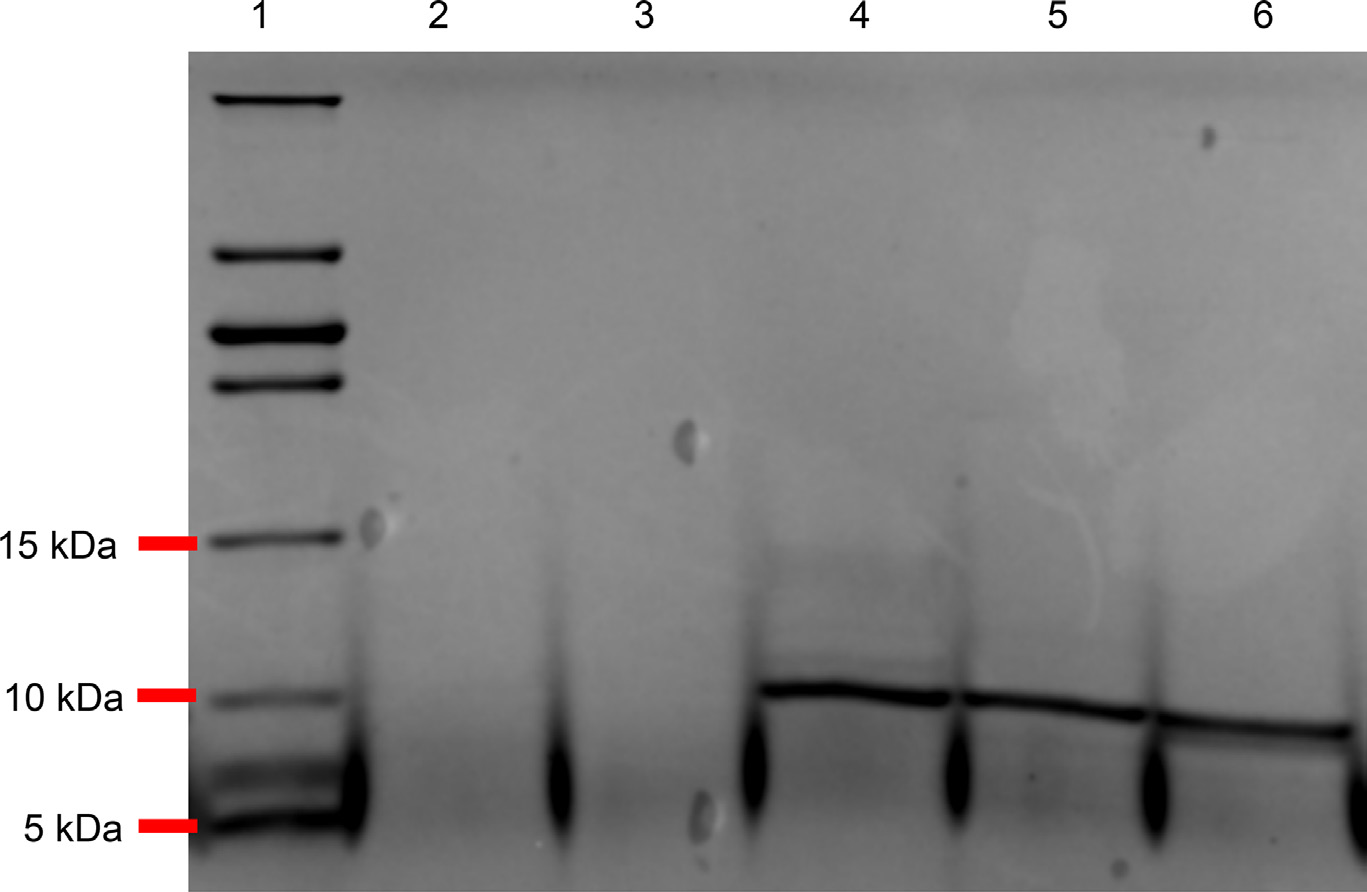
Tris-tricine SDS-PAGE of His-tag cleaved IP_HG88_ digested with protease enzymes. Lane 1: low range protein ladder. Lanes 2–5: digested by protease K, pepsin, chymotrypsin and trypsin, respectively. Lane 6: no digestion (control).

To further characterize the purified IP_HG88_ peptide, the peptide with the His-tag cleaved was subjected to protease digestions. Similar to HG88 CFS, the peptide was resistant to trypsin and chymotrypsin but completely digested by protease K and pepsin (Fig. 6), which corresponded to the observed loss of antibacterial activity (either 100% or 0%) towards *C. perfringens* after treatment with the various proteases.

## Discussion

*B. velezensis* HG88 described in this study exhibited protease activity during the isolate selection and is resistant to heat treatment (80 °C, 10 min). In addition, both the CFS and purified antibacterial peptide (IP_HG88_) from this strain have a strong antimicrobial activity towards *C. perfringens*. The activity was not abolished by heat treatment or with the proteases trypsin and chymotrypsin. Moreover, the antimicrobial activity appears to target *C. perfringens*, but not other bacteria and yeast including the major groups of commensal and beneficial gut bacteria such as *Enterococcus*, *Escherichia coli*, *Bifidobacterium* and *Lactobacillus* [25]. These properties offer good potential for the strain to be used in chicken production in the post-antibiotic era to control NE disease caused by *C. perfringens* (type G). However, further animal studies are required to confirm its efficacy in controlling NE disease and effects on chicken gut health (including the gut microbiota) and growth performance, safe use, product quality and cost-effectiveness in application [12].

**Fig. 7.**
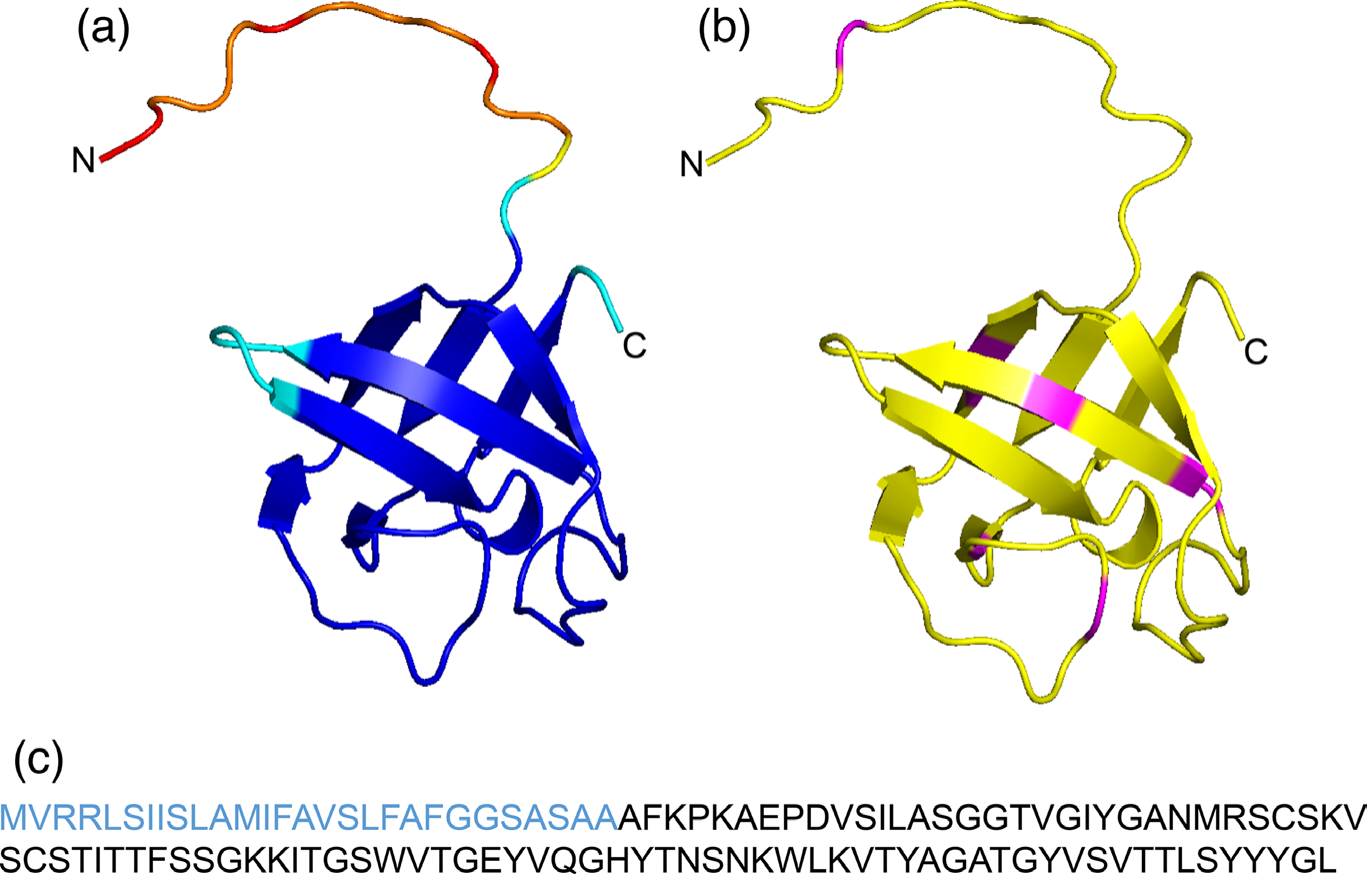
IP_HG88_ primary and predicted tertiary structures. This figure shows the model of the full-length IP_HG88_ protein (including the N-terminal signal peptide) generated by AlphaFold2. (**a**) IP_HG88_ coloured by predicted pLDDT score. Blue shows the highest and red shows the lowest accuracy. (**b**) IP_HG88_ structure showing the predicted trypsin cutting sites (magenta). (**c**) Full-length sequence of IP_HG88_ with the sequence for the signal peptide in blue.

In the present study, purified IP_HG88_ was digested completely by protease K, but not by trypsin. Protease K digests peptide bonds non-specifically at carboxyl groups of aliphatic and aromatic aa, while trypsin cleaves specifically peptide bonds at the C-terminal side of lysine and arginine residues [26]. AlphaFold 2.0 was used to predict the 3D structure of IP_HG88_. The predicted trypsin sites were mapped onto the AlphaFold 2.0 model of IP_HG88_ (Fig. 7), showing that the sites are within the *β*-sheets of the protein, possibly hindering access by trypsin [26].

It is of interest that IP_HG88_ is predicted to be a standalone SH3 domain protein. The protein contains a signal peptide that enables its secretion into the extracellular space. When the signal peptide was replaced by the His-tag at the N-terminal in the recombinant *Escherichia coli*, the protein was detected in the cytoplasm. The purified IP_HG88_ with or without His-tag retained a full antibacterial activity towards *C. perfringens* CP1 with an MBC of ~39–57 µg ml^−1^. IP_HG88_ shares sequence similarity to a partial 28 aa sequence of a peptide (called LC3), which was isolated from *B. subtilis* and inhibited the growth of *Xanthomonas campestris pv. Oryzea*, a rice bacterial blight pathogen [27]. To date, neither further work on LC3 nor its full-length sequence and mechanism of inhibition against *Xanthomonas* has been reported. In addition, LC3 was determined to have a M_W_ of 26.9 kDa [27] that is considerably larger than IP_HG88_ (M_W_ ~9 kDa). Therefore, it is unlikely that LC3 contains only a single SH3 domain like IP_HG88_. No protein with a standalone SH3 domain and minimal other features similar to IP_HG88_ has been reported thus far to have an antibacterial activity, suggesting that IP_HG88_ is a novel antibacterial peptide.

The SH3 domain is a motif found in eukaryotic signal transduction proteins where they mediate protein–protein interactions. In bacteria, the SH3 domains are involved in interactions with the bacterial cell wall and metal ion coordination [28]. Specifically, many peptidoglycan-modifying enzymes contain one or more SH3 domains that anchor the enzymes to peptidoglycan. Peptidoglycan is made up of alternating *N*-acetylglucosamine and *N*-acetylmuramic acid glycan chains that are linked by *β*-1,4-glycosidic linkages. Attached to *N*-acetylmuramic acid is a short peptide usually consisting of four aa residues. The tetrapeptides attached to different *N*-acetylmuramic acids on the glycan chain can be cross-linked, either directly or through a linker peptide. Enzymes involved in cleaving the glycan or peptide chains of peptidoglycan play an important role in cell wall remodelling in bacteria during growth and division [29,30]. Studies of SH3 domains linked to these enzymes suggest that the SH3 domain specifically interacts with the peptide portion of peptidoglycan. For example, lysostaphin, an antimicrobial peptide produced by *Staphylococcus simulans* acts on the peptidoglycan of *Staphylococcus aureus* [28]. The protein has a glycylglycine endopeptidase domain linked to a single SH3 domain. The endopeptidase is responsible for cleaving the pentaglycine cross-link between peptidoglycan tetrapeptides, and the SH3 domain specifically binds to the pentaglycine so that the endopeptidase is targeted to its substrate. The bacterial SH3 domain (commonly referred to as SH3b) is structurally similar to the eukaryotic counterparts with some notable differences. Specifically, there are an extra ~20 aa residues at the N-terminus of SH3b forming 2 *β*-strands that are absent in eukaryotic SH3. In addition, a loop referred to as the RT loop of eukaryotic SH3 is replaced by two *β*-strands (strands 3 and 4) in SH3b [28]. The AlphaFold 2.0 model of IP_HG88_ is similar to canonical SH3b domains. However, the loop between *β*5 and *β*6 (the Src loop) is longer in IP_HG88_ (Fig. 7).

*C. perfringens* also contains peptidoglycan-modifying enzymes with SH3 domains. For example, it has a glucosaminidase containing ten SH3 domains, which can anchor the enzyme to peptidoglycan for remodelling [31]. One possible mechanism of inhibition by IP_HG88_ could therefore involve its binding to peptidoglycan competing for binding sites and thereby inhibiting peptidoglycan-modifying enzymes, such as the aforementioned glucosaminidase. In fact, preliminary results from microscopic examination in the present study revealed a small portion of elongated and filamentous cells of *C. perfringens* CP1 together with some cells that showed cell division defects after the treatment with 46 µg ml^−1^ of His-tag cleaved IP_HG88_ (data not shown). The observations are consistent with defects in cell division due to compromised peptidoglycan remodelling activity [32]. To elucidate the molecular mechanism responsible for the antibacterial activity towards *C. perfringens* and confirm it is not toxic to chickens, further studies are required.

## Abbreviations

ACN: acetonitrile
BHI: Brain Heart Infusion
CFS: cell-free supernatant
DDA: data-dependent acquisition
LB: lysogeny broth
LC-MS: liquid chromotography-mass spectrometry
MBC: minimum bactericidal concentration
MRS: Man–Rogosa–Sharpe
M_W_: molecular weight
NE: necrotic enteritis
pLDDT: predicted local distance difference test
WGS: whole-genome sequencing
